# The value of off-pump coronary artery bypass grafting in the surgery for combined valvular and coronary heart disease

**DOI:** 10.3389/fmed.2024.1451778

**Published:** 2024-11-21

**Authors:** Haokai Qin, Pengrui Si, Kun Hua, Xiubin Yang

**Affiliations:** ^1^Department of Cardiovascular Surgery, Beijing Anzhen Hospital, Capital Medical University, Beijing, China; ^2^Chinese Institutes for Medical Research, Beijing, China

**Keywords:** coronary artery bypass grafting, valve surgery, cardiopulmonary bypass, postoperative outcomes, surgery technique

## Abstract

**Background:**

Combined valve and coronary surgery is a commonly performed surgical technique for treating coexisting valvular and coronary artery disease. This study aims to investigate the effect of reducing the duration of cardiopulmonary bypass by utilizing the off-pump coronary artery bypass grafting (OPCABG) technique on the short-term prognosis of patients.

**Methods:**

In this retrospective cohort study, 884 patients were divided into groups undergoing OPCABG or on-pump CABG combined with valve surgery based on the CABG technique. We evaluated the relationship between the surgical technique and operative mortality, postoperative atrial fibrillation (POAF), stroke, acute kidney failure (AKI), and perioperative myocardial infarction. Propensity score matching and inverse probability weighting (IPTW) were employed to mitigate differences in baseline characteristics between the two groups.

**Results:**

The incidence of POAF and AKI were lower in the OPCABG group after IPTW (POAF: 29.5% vs. 39.5%, *p* = 0.016; AKI: 14.5% vs. 21.2%, *p* = 0.047). OPCABG technique was independently associated with the POAF (adjusted OR: 0.63, 95% CI: 0.44–0.91, *p* = 0.014) and AKI (adjusted OR: 0.63, 95% CI: 0.39–0.98, *p* = 0.049). These results remained even following PSM and IPTW analyses.

**Conclusion:**

The OPCABG technique is associated with reduced occurrences of POAF and AKI in patients undergoing valve and concomitant coronary surgery and can be safely used.

## 1 Introduction

As cardiovascular disease incidence gradually rises, there’s a notable increase in patients with both valvular and coronary artery disease (1). The standard surgical treatment for these cases often recommended as coronary artery bypass grafting (CABG) combined with valve surgery. However, due to the complexity of this procedure, these patients face significantly higher perioperative mortality compared to those undergoing isolated valve surgery or CABG (2). OPCABG was initially proposed for coronary heart disease patients undergoing isolated CABG. Several studies, including the ROOBY trial and the CORONARY trial, have indicated that this technique does not show significant differences in perioperative survival rates compared to traditional on-pump coronary artery bypass grafting (ONCABG) technique; however, it lacks advantages in enhancing long-term survival rates, graft patency, and reducing the incidence of cardiovascular-related adverse events (3–7). Nonetheless, the patient in these studies exclusively underwent isolated CABG surgery, and the effects of this technique in combined valve and coronary surgeries remain under explored.

The primary advantage of OPCABG technique in combined surgeries is its ability to reduce the duration of cardiopulmonary bypass (CPB) to provide better myocardial protection. Some surgeons prefer this technique for patients who need more bridging grafts and for patients with poor target vessel conditions. Current studies have indicated that prolonged CPB time is a major risk factor for perioperative mortality in patients underwent combined surgery (8). However, it remains unclear whether OPCABG technique truly provides any benefit to these patients. Therefore, further research is needed to comprehensively elucidate the impact of this surgical technique.

Our study examined the impact of using OPCABG technique during combined valve and coronary surgery on improving short-term prognosis in patients with both valvular and coronary artery disease in a large cohort.

## 2 Method

### 2.1 Establishing the study populations and surgical methods

In this retrospective study, we enrolled 1,088 patients who underwent CABG combined with valve surgery in Beijing Anzhen Hospital from January 2021 to December 2023. The exclusion criteria are as follows: (1) under 18 years old; (2) underwent emergency surgery; (3) missed postoperative continuous electrocardiography (ECG) records; (4) combined with aortic dissection. Finally, a total of 884 patients were included in our study. This study was approved by the Ethics Committee of Beijing Anzhen Hospital, Capital Medical University (ethics approval number: 2024104X).

Patients were divided into OPCABG plus valve surgery group (OPCABG group, *n* = 173) and ONCABG plus valve surgery group (ONCABG group, *n* = 711) based on whether they underwent CABG under CPB. In the OPCABG group, the distal end of the veins or internal mammary artery was anastomosed to the target coronary artery with the assistance of a tissue stabilizer in the beating heart state, and then routine intubation was performed to establish CPB. For those without aortic valve disease, the ascending aortic root was clamped with a atraumtic lateral wall clamps and used aortic punche to create circular holes, then anastomosed the proximal end of the bridge vessel in aorta (if the bridge vessels were veins) to completed all CABG operations. The ascending aorta was blocked, and the root was perfused with cardioplegia to induce cardiac arrest, and valve replacement or plastic was performed. For those with aortic valve disease, completed the distal anastomotic of bridge vessel, blocked and cut the ascending aorta, perfused cardioplegia through the left and right coronary artery and venous bridge vessel opening, after cardiac arrest, performed valve replacement or plastic, and then anastomosed the proximal end of the bridge vessel to the ascending aorta. In the ONCABG group, established CPB and blocked ascending aorta and perfused the cardioplegia after median sternotomy, then performed CABG and valve surgery under cardiac arrest. After all the process, checked for inactivity of the bleeding, leaved the pericardium and thoracic drainage tube, fixed the sternum, sutured the incision layer by layer.

### 2.2 Data collection and patient outcomes

We recorded the patients’ basic preoperative and postoperative data and medication histories. Operative mortality was defined as any death, regardless of cause, occurring within 30 days after surgery, whether in or out of the hospital (9). Postoperative atrial fibrillation (POAF) was defined as atrial fibrillation lasting longer than 1 h and/or requiring treatment after surgery (10). Postoperative acute kidney injury (AKI) was characterized by a 0.3 mg/dL or 50% increase in serum creatinine from baseline or oliguria (11). Postoperative stroke was defined as a permanent neurologic impairment diagnosed by a neurologist and confirmed by imaging evidence of cerebral artery occlusion (12). Perioperative myocardial infarction (PMI) was defined as the elevation of hsTnI values with the evidence of myocardial ischemia (new Q waves or imaging evidence of myocardial ischemia). All recorded results occurred within 30 days after surgery, but POAF was recorded for only 5 days because continuous postoperative ECG monitoring is usually maintained for only 5 days after surgery. Heart failure was defined according to the Framingham criteria. Other comorbidities were classified according to the International Classification of Diseases, Ninth Revision, and Clinical Modification codes. Preoperative medical treatment statistics included oral medications taken by patients for more than one month prior to surgery, with dosages and regimens determined according to the treatment guidelines outlined in the relevant clinical documentation.

### 2.3 Statistical analysis

Categorical data were compared using Chi-squared tests. The normality distribution test for continuous variables was performed using the Kolmogorov–Smirnov test. Abnormal distributed continuous variables were presented as interquartile ranges, and intergroup comparisons by using Mann–Whitney U test. Missing values were filled by using mean interpolation.

To mitigate selection bias and address potential confounding factors between the groups, we employed propensity score matching (PSM) and inverse probability of treatment weighting (IPTW) analysis to adjust for discrepancies in the baseline characteristics of the patients. PSM was performed using the 1:1 nearest neighbor matching method and optimal matching with a caliper width of 0.3 standard deviations. The following baseline covariates included in the PSM model were age, male, hypertension, diabetes, chronic kidney disease, body mass index, left ventricular ejection fraction troponin I and brain natriuretic peptide. These covariates were selected for inclusion in the models based on the fact that they are commonly associated risk factors for poor prognosis in cardiac surgery and after incorporating these covariates, no model-fitting issues were identified, and a satisfactory balance between treatment groups was achieved following weighting, with no extreme weights observed. These covariates were also included in the multivariate logistic regression model. The propensity scores were converted to IPTW with OPCABG patients having a weighting of 1. Logistic regression was then used on PSM and IPTW models to estimate odds ratios (ORs) and 95% confidence intervals (CIs) for independent correlations between OPCABG groups and perioperative outcomes.

Statistical analyses were performed with SPSS (IBM SPSS Statistics for Windows, version 26.0) and R software (version 4.3.3, R Foundation for Statistical Computing). The statistical significance level was set at two-tailed *p* < 0.05.

## 3 Results

### 3.1 Patient baseline characteristics

**Figure 1:**
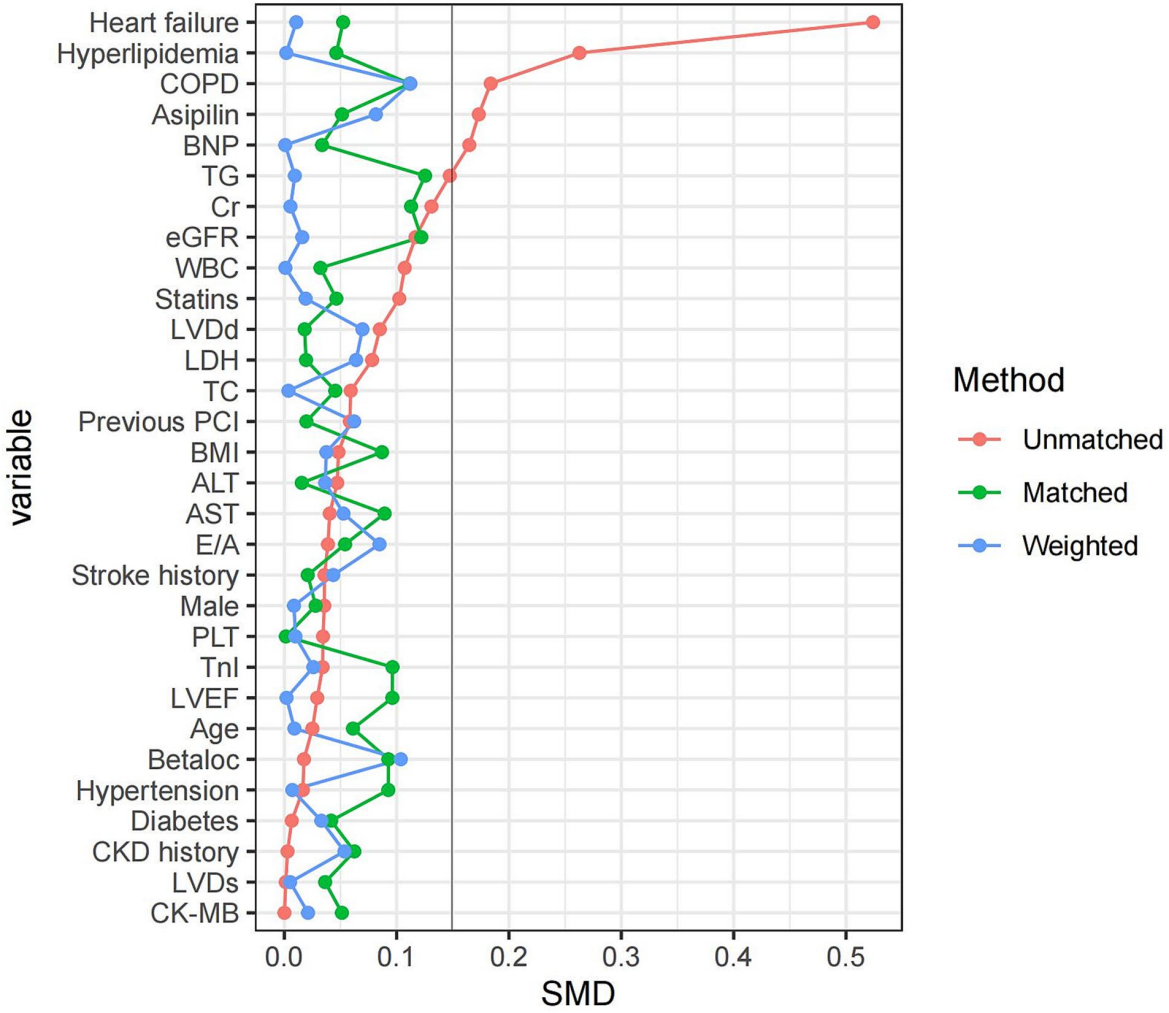
Covariate balance plot for assessing balance between OPCABG group and ONCABG group after PSM and IPTW.

Out of the 884 patients included in this study, 76.2% were male. The baseline characteristics of the patients in each group are shown in Table 1. There was a significant difference between the OPCABG group and the ONCABG group in history of hyperlipidemia, heart failure, aspirin usage (*p* < 0.05). The OPCABG group had a higher number of graft (*p* < 0.01). After PSM and IPTW, differences in baseline data were significantly controlled (Figure 1; Supplementary Table S1; Supplementary Table S2).

**Table 1:** Baseline characteristics between OPCABG group and ONCABG group.

	OPCABG (*n* = 173)	ONCABG (*n* = 711)	*p* value
Demographics			
Age, years	63 [56, 69]	64 [57, 68]	0.85
Male (%)	134 (77.5)	540 (75.9)	0.75
BMI, kg/m^2^	25.18 [23.24,27.15]	25.18 [23.16, 26.98]	0.858
Comorbidity			
Hypertension (%)	83 (48.0)	347 (48.8)	0.912
Diabetes (%)	37 (21.4)	154 (21.7)	1.00
COPD (%)	10 (5.8)	77 (10.8)	0.063
Hyperlipidemia (%)	81 (46.8)	242 (34.0)	0.002
CKD (%)	2 (1.2)	8 (1.10)	1.00
PCI history (%)	17 (9.8)	58 (8.2)	0.579
Stroke history (%)	15 (8.7)	69 (9.7)	0.786
Heart failure (%)	48 (27.7)	374 (52.6)	0.001
Preoperative medical treatment			
Statins (%)	94 (54.3)	350 (49.2)	0.263
Aspirin (%)	51 (29.5)	156 (21.9)	0.046
Betaloc (%)	80 (46.2)	335 (47.1)	0.903
Preoperative laboratory data			
TG, mmol/L	1.42 [1.05, 1.6]	1.40 [1.04, 1.73]	0.631
TC, mmol/L	4.17 [3.48, 4.75]	4.17 [3.53, 4.76]	0.769
Cr, μmol/L	78.6 [70.3,90.2]	80.30 [70.05, 94.75]	0.327
ALT, U/L	17 [12,27]	17 [12, 27]	0.666
AST, U/L	19 [16,30]	20 [16, 33.5]	0.311
PLT, 10^9/L	160 [109,217]	158 [110,204]	0.407
WBC, 10^9/L	7.42 [5.85, 9.78]	7.69 [5.94, 10.59]	0.204
eGFR, mL/min	86.6 [72.29,96.63]	84.74 [69.07, 95.87]	0.173
LDH, U/L	195 [170,230]	200 [173,252]	0.102
CK-MB, ng/mL	1.80 [1.3, 2.9]	2 [1.4, 3.2]	0.127
TnI, ng/mL	1.9 [1.1, 2.73]	2.10 [1.20, 2.8]	0.222
BNP, pg/mL	244 [116,492.87]	295 [114,534]	0.196
Preoperative echocardiographic data			
LVEF, %	58 [51,62]	57 [51, 63]	0.92
E/A ratio	1.23 [0.73, 1.45]	1.21 [0.75, 1.42]	0.959
LVDD, mm	51 [47,56]	50 [45, 56]	0.149
LVDS, mm	35 [30,39]	34 [30, 40]	0.653
CABG data			
LIMA usage (%)	48 (27.7)	178 (25.0)	0.525
Graft number	3.00 [2.00, 4.00]	2.00 [1.00, 3.00]	<0.001
Types of valve surgery			
Single valve surgery			
Mitral valve replacement (%)	36 (20.8)	101 (14.2)	0.042
Mitral valve repair (%)	24 (13.9)	109 (15.3)	0.717
Aortic valve replacement (%)	44 (25.4)	219 (30.8)	0.196
Aortic valve repair (%)	1 (0.6)	3 (0.4)	1
Tricuspid valve replacement (%)	0 (0)	0 (0)	1
Tricuspid valve repair (%)	5 (2.9)	19 (2.7)	1
Double valve surgery			
Double valve replacement (%)	3 (1.7)	13 (1.8)	1
Double valve repair (%)	1 (0.6)	2 (0.3)	1
Combined repair and replacement (%)	29 (16.8)	93 (13.1)	0.256
Triple valve surgery			
Triple valve replacement (%)	0 (0.0)	1 (0.1)	1
Triple valve repair (%)	0 (0.0)	2 (0.3)	1
Combined repair and replacement (%)	30 (17.3)	149 (21.0)	0.339
Mechanical valve (%)	55 (31.8)	282 (39.7)	0.068
Bioprosthetic valve (%)	87 (50.3)	298 (41.9)	0.056

Data are presented as median [25th-75th percentiles] or *n* (%). BMI, body mass index; COPD, chronic obstructive pulmonary disease; CKD, chronic kidney disease; PCI, percutaneous coronary intervention; TG, triglyceride; TC, total cholesterol; Cr, creatinine; ALT, alanine aminotransferase; AST aspartate transaminase; LDH, lactate dehydrogenase; PLT, platelet; WBC, white blood cell; eGFR, estimated glomerular filtration rate; LDH, lactate dehydrogenase; CK-MB, creatine kinase MB; TnI, troponin I; BNP, brain natriuretic peptide; LVEF, left ventricular ejection fractions; E/A, ratio early to late diastolic transmitral flow velocity; LVDD, left ventricular end-diastolic dimension; LVDS, left ventricular end-systolic dimension; CABG, coronary artery bypass grafting; LIMA, left internal mammary artery.

### 3.2 Comparing perioperative outcomes between OPCABG group and ONCABG group

After IPTW analysis, CPB time and crossclamp time were significantly shorter in the OPCABG group (*p* < 0.001). Additionally, the OPCABG group had a lower incidence of POAF (IPTW: 29.5% vs. 39.5%, *p* = 0.015) and AKI (IPTW: 14.5% vs. 21.2%, *p* = 0.049) compared to the ONCABG group, though there was no difference in the incidence of operative mortality, stroke, AMI, and PMI. Furthermore, we compared changes in myocardial enzymes CK-MB and hsTnI over the three days following surgery. The results indicated no statistically significant differences between the two groups, despite the levels being lower in the OPCABG group (Table 2; Supplementary Table S3).

**Table 2:** Postoperative outcomes between OPCABG group and ONCABG group after IPTW.

	OPCABG (*n* = 173)	ONCABG (*n* = 172.95)	*p* value
Operative mortality (%)	9 (5.2)	6.7 (3.9)	0.441
POAF (%)	51 (29.5)	68.3 (39.5)	0.016
Stroke (%)	4 (2.3)	5.2 (3.0)	0.625
AKI (%)	25 (14.5)	36.7 (21.2)	0.047
PMI (%)	2 (1.2)	1.5 (0.8)	0.698
CK-MB, ng/mL			
24 h	68.85 [42.85,97.75]	66 [42.11,104]	0.939
48 h	47 [27.70,78]	45.58 [25.51,78.34]	0.586
72 h	27.5 [10.03, 54.02]	22.84 [10.30, 50.70]	0.566
hsTnI, ng/mL			
24 h	6.14 [3.62,9.55]	6.19[3.41,10.2]	0.425
48 h	3.65 [2.05,6.42]	3.84[1.95,6.43]	0.655
72 h	2.1 [0.98,3.51]	2.89[1.13, 3.51]	0.255
Operation time, hours	6 [5, 7]	6 [5, 7]	0.536
Crossclamp time, min	86 [69, 121.75]	113 [93, 139]	<0.001
CPB time, min	160 [119.25, 188.75]	167 [142, 203.32]	<0.001
Ventilation time, hours	24.75 [17.62, 54.38]	23.5 [17.5, 48.36]	0.283
LOS, days	7.5 [6, 10]	8 [6, 10]	0.899
ICU time of stay, hours	41.5 [19, 67.75]	34 [18, 67]	0.267
IABP (%)	13.0 (7.5)	15.6 (9)	0.532
ECMO (%)	2.0 (1.2)	3.0 (1.7)	0.599

Data are presented as median [25th–75th percentiles] or *n* (%). POAF, postoperative atrial fibrillation; AKI, acute kidney injury; PMI, postoperative myocardial infarction; CK-MB, creatine kinase MB; hsTnI, high-sensitivity troponin I; CPB, cardiopulmonary bypass; LOS, length of stay; ICU, intensive care unit; IABP, intra-aortic ballon pump; ECMO, extracorporeal membrane oxygenator.

Logistic regression analysis revealed that the OPCABG technique was significantly associated with a reduced occurrence of POAF (adjusted OR: 0.63, 95% CI: 0.44–0.91, *p* = 0.014) and AKI (adjusted OR: 0.63, 95% CI: 0.39–0.98, *p* = 0.049). However, it was not associated with operative mortality (adjusted OR: 1.35, 95% CI: 0.58–2.91, *p* = 0.460), stroke (adjusted OR: 0.74, 95% CI: 0.21–2.01, *p* = 0.592), or PMI (adjusted OR: 1.17, 95% CI: 0.57–2.22, *p* = 0.649). These results remained consistent after PSM and IPTW adjustments (Table 3).

**Table 3:** The ORs for postoperative outcomes in OPCABG group.

Methods	Unadjusted		Multivariable		Matched		Weighted	
	OR (95%CI)	*p* value	OR (95%CI)	*p* value	OR (95%CI)	*p* value	OR (95%CI)	*p* value
POAF	0.64 (0.44, 0.91)	0.015	0.63 (0.44, 0.91)	0.014	0.62 (0.39, 0.96)	0.033	0.64 (0.48, 0.85)	0.002
Perioperative mortality	1.34 (0.59, 2.79)	0.458	1.35 (0.58, 2.91)	0.460	1.13 (0.42, 3.08)	0.804	1.36 (0.72, 2.61)	0.351
Stroke	0.78 (0.22, 2.08)	0.649	0.74 (0.21, 2.01)	0.592	0.56 (0.14, 1.89)	0.364	0.76 (0.33, 1.74)	0.523
AKI	0.63 (0.39, 0.99)	0.051	0.63 (0.39, 0.98)	0.049	0.58 (0.33, 0.99)	0.048	0.63 (0.44, 0.89)	0.009
PMI	1.16 (0.57, 2.18)	0.665	1.17 (0.57, 2.22)	0.649	1.00 (0.43, 2.31)	1.000	1.16 (0.68, 2.00)	0.584

POAF, postoperative atrial fibrillation; AKI, acute kidney failure; PMI, perioperative myocardial infarction; OR, odds ratio; CI, confidence intervals.

### 3.3 Subgroup analysis

**Figure 2:**
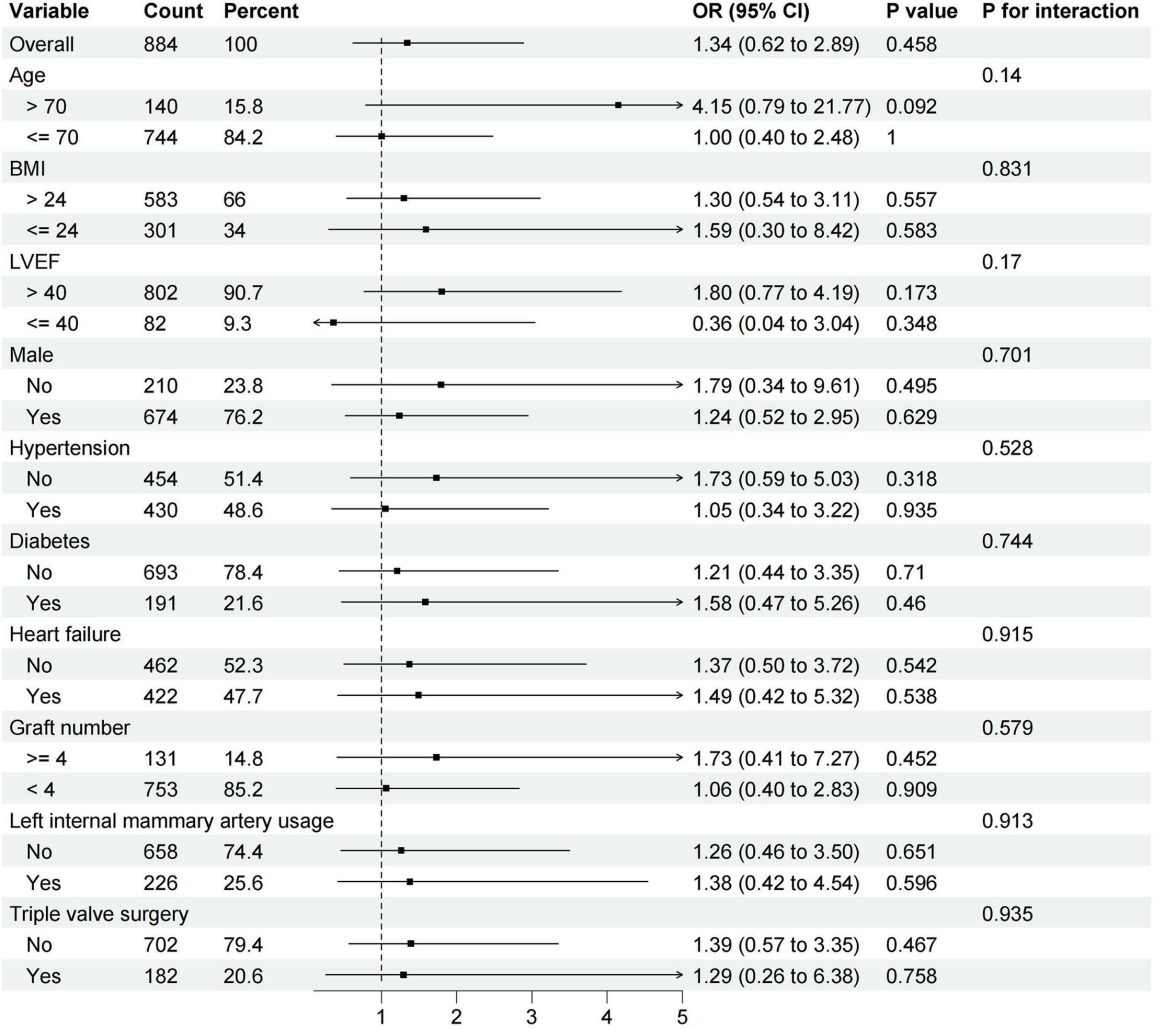
Difference in the incidence of operative mortality according to major subgroups of interest.

Subgroup analysis defined by age, male, BMI, LVEF, hypertension, diabetes, heart failure, graft number, left internal mammary artery usage and triple valve surgery between OPCABG group and ONCABG were performed. In different subgroups, we did not observe an association between the OPCABG technique and operative mortality (Figure 2).

## 4 Discussion

Combined valvular and coronary heart disease is a serious cardiac disease often requiring valve replacement or plasty combined with CABG, with CPB being an indispensable part of the process. CPB has been used in cardiac surgery for over 60 years, and through extensive development, it has matured significantly. The use of cardioplegia along with CPB has enabled the performance of complex cardiac surgical procedures (13). However, CPB still poses several problems and can lead to adverse outcomes. Substantial evidence from various studies on extracorporeal circulation indicates that CPB induces a systemic inflammatory response and increases oxidative stress (14–16). Additionally, the contact of blood with the inner surface of the CPB tubing activates the coagulation cascade, resulting in coagulation disorders (17), which significantly affect the incidence of postoperative complications.

To minimize the adverse effects of CPB, some new methods such as the miniaturized cardiopulmonary bypass system (18), low-level light therapy (19, 20), and anesthetics with anti-inflammatory effects (21, 22) have been developed. However, due to uncertain results or early developmental stages, these methods are rarely used in clinical. A simpler and more feasible approach has been proposed: reducing the duration of CPB through OPCABG technique, thus improving short-term patient prognosis (23). Data from our center also show that surgeons favor the OPCABG procedure for a subset of patients who need more bridging vascular grafts because the procedure time is usually longer in this subset of patients, and the reduced CPB time of OPCABG may provide better myocardial protection. Although some relevant studies exist, most have small sample sizes. Our study addresses this shortcoming.

The results of our research showed that the OPCABG group did not exhibit significant differences in the incidence of perioperative death, stroke, and infection compared to the ONCABG group. However, the OPCABG technique was more effective in reducing the incidence of POAF and AKI. These satisfactory outcomes indicate that this technique is both safe and possible in combined valve and coronary surgery.

Inflammation and myocardial ischemia are key mechanisms that promote the development of POAF (24). In our research, the incidence of POAF was significantly lower in the OPCABG group, which may be attributed to the fact that OPCABG technique shortens the duration of cardiac arrest and reduces the damage caused by myocardial ischemia (25), which in turn attenuates the inflammatory response caused by myocardial ischemia. Some studies have confirmed the damaging effects of CPB on the kidneys (26). In our research, the incidence of postoperative AKI was lower in the OPCABG, which is related to the fact that hypothermia and the increase in systemic vascular resistance during prolonged CPB make the renal vasoconstriction, which in turn results in renal ischemia and injury (27). In addition, a shorter duration of CPB will similarly reduce renal damage from inflammatory factors.

The concept of minimizing myocardial injury by shortening CPB time was originally proposed for isolated coronary artery bypass grafting (28). We selected CK-MB and hsTnI, which reflect the degree of myocardial damage, to determine the recovery of the heart in the two groups of patients after surgery. As a result, patients in the OPCABG group had lower levels of these two cardiac enzymes three days after surgery, but there was no statistically significant difference, which is also the same as the results of Rogers et al. (18). In addition to shortening the duration of CPB through surgical techniques, the application of long-acting cardioplegia may can also have a beneficial effect on myocardial protection. Use of long-acting cardioplegia can provide a practical advantage as it allows the surgeon a longer arrest period before a subsequent dose (29, 30).

The greatest advantage of applying the OPCABG technique to CABG combined with valve surgery is that it shortens the duration of CPB to reduces myocardial injury and inflammatory response. Although our research results indicate that the OPCABG technique is more beneficial for patients’ short-term prognosis, we suggested that surgeons should also consider individual patient conditions when selecting surgical options.

## 5 Limitation

Since this is a retrospective study, we were unable to collect all unknown confounding factors especially some intraoperative variables, such as type of cardioplegia and the details of CABG were not included in the design of this study, which may have led to biased results. Due to the retrospective nature, OPCABG might be more likely to be performed in patients with more complicated coronary anatomy, and this may lead to selection bias. Since only patients who signed the informed consent form and agreed to participant in this research were counted, this may also affect the accuracy of the conclusions. In addition, this study did not evaluate the effect of the OPCABG technique on long-term prognosis.

## 6 Conclusion

In this retrospective research, we found that the OPCABG technique may be able to be safely applied to CABG combined valve surgery. Furthermore, OPCABG technique is more beneficial for patients’ short-term prognosis and can reduce the incidence of POAF and AKI.

## Data Availability

The raw data supporting the conclusions of this article will be made available by the authors, without undue reservation.

## References

[1] Buth KJ Gainer RA Legare JF Hirsch GM. The changing face of cardiac surgery: practice patterns and outcomes 2001-2010. Can J Cardiol. (2014) 30:224–30. doi: 10.1016/j.cjca.2013.10.020 24373760

[2] Hannan EL Wu C Bennett EV Carlson RE Culliford AT Gold JP. Risk index for predicting in-hospital mortality for cardiac valve surgery. Ann Thorac Surg. (2007) 83:921–9. doi: 10.1016/j.athoracsur.2006.09.051 17307434

[3] Yang L Lin S Zhang H Gu D Chen S Shi Y. Long-term graft patency after off-pump and on-pump coronary artery bypass: a CORONARY trial cohort. Ann Thorac Surg. (2020) 110:2055–61. doi: 10.1016/j.athoracsur.2020.03.053, PMID: 32339504

[4] Shroyer AL Grover FL Hattler B Collins JF GO MD Kozora E. On-pump versus off-pump coronary-artery bypass surgery. N Engl J Med. (2009) 361:1827–37. doi: 10.1056/NEJMoa0902905 19890125

[5] Shroyer AL Hattler B Wagner TH Collins JF Baltz JH Quin JA. Five-year outcomes after on-pump and off-pump coronary-artery bypass. N Engl J Med. (2017) 377:623–32. doi: 10.1056/NEJMoa1614341 28813218

[6] Zhou Z Fu G Feng K Huang S Chen G Liang M. Randomized evidence on graft patency after off-pump versus on-pump coronary artery bypass grafting: an updated meta-analysis. Int J Surg. (2022) 98:106212. doi: 10.1016/j.ijsu.2021.106212, PMID: 35041977

[7] Zhou Z Liang M Zhuang X Liu M Fu G Liu Q. Long-term outcomes after on-pump vs off-pump coronary artery bypass grafting for ischemic cardiomyopathy. Ann Thorac Surg. (2023) 115:1421–8. doi: 10.1016/j.athoracsur.2021.12.063 35085524

[8] Fudulu DP Layton GR Nguyen B Sinha S Dimagli A Guida G. Trends and outcomes of concomitant aortic valve replacement and coronary artery bypass grafting in the UK and a survey of practices. Eur J Cardiothorac Surg. (2023) 64:ezad259. doi: 10.1093/ejcts/ezad259 37462523 PMC10580967

[9] Jacobs JP Mavroudis C Jacobs ML Maruszewski B Tchervenkov CI Lacour-Gayet FG. What is operative mortality? Defining death in a surgical registry database: a report of the STS congenital database taskforce and the joint EACTS-STS congenital database committee. Ann Thorac Surg. (2006) 81:1937–41. doi: 10.1016/j.athoracsur.2005.11.063, PMID: 16631716

[10] Society of Thoracic Surgeons. (2019). Adult cardiac surgery database collection. Available online at: https://www.sts.org/registries-research-center/sts-national-database/adult-cardiac-surgery-database/data-collection (Accessed May 13, 2019)

[11] Brown JR Baker RA Shore-Lesserson L Fox AA Mongero LB Lobdell KW. The Society of Thoracic Surgeons/Society of Cardiovascular Anesthesiologists/American Society for Extracorporeal Technology Clinical Practice Guidelines for the prevention of adult cardiac surgery-associated acute kidney injury. Anesth Analg. (2023) 136:176–84. doi: 10.1213/ANE.0000000000006286, PMID: 36534719

[12] Hicks KA Mahaffey KW Mehran R Nissen SE Wiviott SD Dunn B. 2017 Cardiovascular and stroke endpoint definitions for clinical Trials. Circulation. (2018) 137:961–72. doi: 10.1161/CIRCULATIONAHA.117.033502, PMID: 29483172

[13] Hessel EA 2nd. What's new in cardiopulmonary bypass. J Cardiothorac Vasc Anesth. (2019) 33:2296–326. doi: 10.1053/j.jvca.2019.01.039, PMID: 30928282

[14] Kowalik MM Lango R Siondalski P Chmara M Brzeziński M Lewandowski K. Clinical, biochemical and genetic risk factors for 30-day and 5-year mortality in 518 adult patients subjected to cardiopulmonary bypass during cardiac surgery – the INFLACOR study. Acta Biochim Pol. (2018) 65:241–50. doi: 10.18388/abp.2017_2361, PMID: 29694446

[15] Long DM Jenkins E Griffith K. Perfusionist techniques of reducing acute kidney injury following cardiopulmonary bypass: an evidence-based review. Perfusion. (2015) 30:25–32. doi: 10.1177/0267659114544395, PMID: 25073949

[16] Karu I Taal G Zilmer K Pruunsild C Starkopf J Zilmer M. Inflammatory/oxidative stress during the first week after different types of cardiac surgery. Scand Cardiovasc J. (2010) 44:119–24. doi: 10.3109/14017430903490981, PMID: 20141341

[17] Doyle AJ Hunt BJ. Current understanding of how extracorporeal membrane oxygenators activate Haemostasis and other blood components. Front Med. (2018) 5:352. doi: 10.3389/fmed.2018.00352, PMID: 30619862 PMC6299009

[18] Anastasiadis K Murkin J Antonitsis P Bauer A Ranucci M Gygax E. Use of minimal invasive extracorporeal circulation in cardiac surgery: principles, definitions and potential benefits. A position paper from the Minimal invasive Extra-Corporeal Technologies international Society (MiECTiS). Interact Cardiovasc Thorac Surg. (2016) 22:647–62. 26819269 10.1093/icvts/ivv380 PMC4892134

[19] Walski T Drohomirecka A Bujok J Czerski A Wąż G Trochanowska-Pauk N. Low-level light therapy protects red blood cells against oxidative stress and hemolysis during extracorporeal circulation. Front Physiol. (2018) 9:647. doi: 10.3389/fphys.2018.00647, PMID: 29904353 PMC5991292

[20] Drohomirecka A Iwaszko A Walski T Pliszczak-Król A Wąż G Graczyk S. Low-level light therapy reduces platelet destruction during extracorporeal circulation. Sci Rep. (2018) 8:16963. doi: 10.1038/s41598-018-35311-9, PMID: 30446721 PMC6240032

[21] Hall R. Identification of inflammatory mediators and their modulation by strategies for the management of the systemic inflammatory response during cardiac surgery. J Cardiothorac Vasc Anesth. (2013) 27:983–1033. doi: 10.1053/j.jvca.2012.09.013 23276596

[22] Bulow NM Colpo E Pereira RP Correa EF Waczuk EP Duarte MF. Dexmedetomidine decreases the inflammatory response to myocardial surgery under mini-cardiopulmonary bypass. Braz J Med Biol Res. (2016) 49:e4646. doi: 10.1590/1414-431X20154646, PMID: 26909786 PMC4792505

[23] Rogers CA Capoun R Scott LJ Taylor J Jain A Angelini GD. Shortening cardioplegic arrest time in patients undergoing combined coronary and valve surgery: results from a multicentre randomized controlled trial: the SCAT trial. Eur J Cardiothorac Surg. (2017) 52:288–96. doi: 10.1093/ejcts/ezx087, PMID: 28444178 PMC5848808

[24] Gaudino M Di Franco A Rong LQ Piccini J Mack M. Postoperative atrial fibrillation: from mechanisms to treatment. Eur Heart J. (2023) 44:1020–39. doi: 10.1093/eurheartj/ehad019 36721960 PMC10226752

[25] Tchervenkov CI Wynands JE Symes JF Malcolm ID Dobell AR Morin JE. Persistent atrial activity during cardioplegic arrest: a possible factor in the etiology of postoperative supraventricular tachyarrhythmias. Ann Thorac Surg. (1983) 36:437–43. doi: 10.1016/S0003-4975(10)60484-5 6605126

[26] Pontes JCDV da Silva GVR Benfatti RA Machado NP Pontelli R Pontes ERJC. Fatores de risco no desenvolvimento de insuficiência renal aguda após cirurgia de revascularização miocárdica com CEC. Braz J Cardiovasc Surg. (2007) 22:484–90. doi: 10.1590/S0102-76382007000400016, PMID: 18488117

[27] Soares LC Ribas D Spring R Silva JM Miyague NI. Perfil clínico da resposta inflamatória sistêmica após cirurgia cardíaca pediátrica com circulação extracorpórea [clinical profile of systemic inflammatory response after pediatric cardiac surgery with cardiopulmonary bypass]. Arq bras Cardiol. (2010) 94:127–33. doi: 10.1590/S0066-782X2010000100019 20414536

[28] Murphy GJ Ascione R Angelini GD. Coronary artery bypass grafting on the beating heart: surgical revascularization for the next decade? Eur Heart J. (2004) 25:2077–85. doi: 10.1016/j.ehj.2004.09.022, PMID: 15571822

[29] Guim GS Wah Hoon CG Lim CA Chay-Nancy HS Li Ler AA Lim QX. Use of del Nido Cardioplegia for adult heart surgery: how Long is not too Long? J Extra Corpor Technol. (2020) 52:272–8. doi: 10.1051/ject/202052272 33343029 PMC7728503

[30] Garcia-Suarez J Garcia-Fernandez J Martinez Lopez D Reques L Sanz S Carballo D. Clinical impact of del Nido cardioplegia in adult cardiac surgery: a prospective randomized trial. J Thorac Cardiovasc Surg. (2023) 166:1458–67. doi: 10.1016/j.jtcvs.2022.01.044, PMID: 35279289

