# Supplementary material for: The value of off-pump coronary artery bypass grafting in the surgery for combined valvular and coronary heart disease
Source: Front Med (Lausanne). 2024 Nov 21;11:1451778. doi: 10.3389/fmed.2024.1451778 (PMC11617180; doi:10.3389/fmed.2024.1451778)
Supplement: Supplementary file 1 [file Data_Sheet_1.docx]

Table S1. Baseline characteristics between OPCABG group and ONCABG group after 1:1 PSM

| 1:1 PSM | | | | |
| --- | --- | --- | --- | --- |
|  | | OPCABG  (n=173) | ONCABG  (n=173) | p value |
| Demographics | |  |  |  |
| Age, years | | 63.00 [56.00, 69.00] | 62.00 [55.00, 67.00] | 0.338 |
| Male (%) | | 134 (77.5) | 140 (80.9) | 0.508 |
| BMI, kg/m^2^ | | 25.18 [23.24, 27.15] | 25.18 [23.34, 26.64] | 0.965 |
| **Comorbidity** | | | | |
| Hypertension (%) | | 83 (48.0) | 79 (45.7) | 0.747 |
| Diabetes (%) | | 37 (21.4) | 40 (23.1) | 0.796 |
| COPD (%) | | 10 (5.8) | 19 (11.0) | 0.121 |
| Hyperlipidemia (%) | | 81 (46.8) | 61 (35.3) | 0.038 |
| CKD (%) | | 2 (1.2) | 4 (2.3) | 0.68 |
| PCI history (%) | | 17 (9.8) | 17 (9.8) | 1.00 |
| Stroke history (%) | | 15 (8.7) | 28 (16.2) | 0.051 |
| Heart failure (%) | | 48 (27.7) | 96 (55.5) | 0.001 |
| **Preoperative medical treatment** | | | | |
| Statins (%) | | 94 (54.3) | 88 (50.9) | 0.59 |
| Aspirin (%) | | 51 (29.5) | 35 (20.2) | 0.062 |
| Betaloc (%) | | 80 (46.2) | 85 (49.1) | 0.667 |
| **Preoperative laboratory data** | | | | |
| TG, mmol/L | | 1.42 [1.05, 1.60] | 1.47 [1.03, 1.80] | 0.514 |
| TC, mmol/L | | 4.17 [3.48, 4.75] | 4.09 [3.25, 4.50] | 0.132 |
| Cr, μmol/L | | 78.60 [70.30, 90.20] | 78.40 [70.80, 92.90] | 0.619 |
| ALT, U/L | | 17.00 [12.00, 27.00] | 16.00 [12.00, 27.00] | 0.463 |
| AST, U/L | | 19.00 [16.00, 30.00] | 20.00 [16.00, 34.00] | 0.416 |
| PLT, 10^9/L | | 160.00 [109.00, 217.00] | 167.00 [118.00, 214.00] | 0.513 |
| WBC, 10^9/L | | 7.42 [5.85, 9.78] | 8.03 [6.10, 10.69] | 0.089 |
| eGFR, ml/min | | 86.60 [72.29, 96.63] | 86.32 [69.84, 97.75] | 0.888 |
| LDH, U/L | | 195.00 [170.00, 230.00] | 199.00 [171.00, 245.00] | 0.349 |
| CK-MB, ng/ml | | 1.80 [1.30, 2.90] | 2.00 [1.40, 3.50] | 0.111 |
| TnI, ng/ml | | 2.05[1.33, 2.73] | 1.80[1.10, 2.73] | 0.185 |
| BNP, pg/ml | | 244.00 [116.00, 492.87] | 268.00 [106.00, 492.87] | 0.885 |
| **Preoperative echocardiographic data** | | | | |
| LVEF, % | | 58.00 [51.00, 62.00] | 56.00 [52.00, 62.00] | 0.574 |
| E/A ratio | | 1.23 [0.73, 1.45] | 1.24 [0.78, 1.52] | 0.499 |
| LVDD, mm | | 51.00 [47.00, 56.00] | 50.00 [45.00, 56.00] | 0.307 |
| LVDS, mm | | 35.00 [30.00, 39.00] | 34.00 [30.00, 40.00] | 0.613 |
| **CABG data** | | | | |
| **LIMA usage (%)** | | 48 (27.7) | 46 (26.6) | 0.904 |
| **Graft number** | | 3.00 [2.00, 4.00] | 2.00 [1.00, 3.00] | <0.001 |
| **Types of valve surgery** | | | | |
| **Single valve surgery** | |  |  |  |
|  | **Mitral valve replacement** (%) | 36 (20.8) | 24 (13.9) | 0.118 |
|  | **Mitral valve repair** (%) | 24 (13.9) | 27 (15.6) | 0.762 |
|  | **Aortic valve replacement** (%) | 44 (25.4) | 54 (31.2) | 0.283 |
|  | **Aortic valve repair** (%) | 1 (0.6) | 0 (0.0) | 1 |
|  | **Tricuspid valve replacement** (%) | 0 (0.0) | 0 (0.0) | 1 |
|  | **Tricuspid valve repair** (%) | 5 (2.9) | 2 (1.2) | 0.445 |
| **Double valve surgery** | |  |  |  |
|  | **Double valve replacement** (%) | 3 (1.7) | 4 (2.3) | 1 |
|  | **Double valve repair** (%) | 1 (0.6) | 1 (0.6) | 1 |
|  | **Combined**  **repair and replacement** (%) | 29 (16.8) | 26 (15.0) | 0.769 |
| **Triple valve surgery** | |  |  |  |
|  | **Triple valve replacement** (%) | 0 (0.0) | 0 (0.0) | 1 |
|  | **Triple valve repair** (%) | 0 (0.0) | 0 (0.0) | 1 |
|  | **Combined**  **repair and replacement** (%) | 30 (17.3) | 35 (20.2) | 0.582 |
| **Mechanical valve** (%) | | 55 (31.8) | 63 (36.4) | 0.427 |
| **Bioprosthetic valve** (%) | | 87 (50.3) | 81 (46.8) | 0.591 |

Data are presented as median [25th-75th percentiles] or n (%). BMI, body mass index; COPD, chronic obstructive pulmonary disease; CKD, chronic kidney disease; PCI, percutaneous coronary intervention; TG, triglyceride; TC, total cholesterol; Cr, creatinine; ALT, alanine aminotransferase; AST aspartate transaminase; LDH, lactate dehydrogenase; PLT, platelet; WBC, white blood cell; eGFR, estimated glomerular filtration rate; LDH, lactate dehydrogenase; CK-MB, creatine kinase MB; TnI, troponin I; BNP, brain natriuretic peptide; LVEF, left ventricular ejection fractions; E/A, ratio early to late diastolic transmitral flow velocity; LVDD, left ventricular end-diastolic dimension; LVDS, left ventricular end-systolic dimension; CABG, coronary artery bypass grafting; LIMA, left internal mammary artery.

Table S2. Baseline characteristics between OPCABG group and ONCABG group after IPTW

| IPTW | | | | |
| --- | --- | --- | --- | --- |
|  | | OPCABG  (n=173) | ONCABG  (n=172.95) | p value |
| Demographics | |  |  |  |
| Age, years | | 63 [56, 69] | 63 [57, 68] | 0.853 |
| Male (%) | | 134 (77.5) | 134 (77.5) | 0.998 |
| BMI, kg/m^2^ | | 25.18[23.23, 27.12] | 25.18[23.01, 26.81] | 0.776 |
| **Comorbidity** | | | | |
| Hypertension (%) | | 83 (48) | 83.3 (48.2) | 0.965 |
| Diabetes (%) | | 37 (21.4) | 36.8 (21.3) | 0.979 |
| COPD (%) | | 10 (5.8) | 17.8 (10.3) | 0.068 |
| Hyperlipidemia (%) | | 81 (46.8) | 59.9 (34.7) | 0.003 |
| CKD (%) | | 2 (1.2) | 2.1 (1.2) | 0.963 |
| PCI history (%) | | 17 (9.8) | 14 (8.1) | 0.463 |
| Stroke history (%) | | 15 (8.7) | 16.8 (9.7) | 0.676 |
| Heart failure (%) | | 48 (27.7) | 91.2 (52.7) | 0.001 |
| **Preoperative medical treatment** | | | | |
| Statins (%) | | 94 (54.3) | 85.9 (49.7) | 0.272 |
| Aspirin (%) | | 51 (29.5) | 38.2 (22.1) | 0.041 |
| Betaloc (%) | | 80 (46.2) | 81.6 (47.2) | 0.82 |
| **Preoperative laboratory data** | | | | |
| TG, mmol/L | | 1.42 [1.05, 1.59] | 1.41 [1.04, 1.75] | 0.472 |
| TC, mmol/L | | 4.17 [3.47, 4.74] | 4.17 [3.53, 4.76] | 0.71 |
| Cr, μmol/L | | 78.6 [70.23, 90.17] | 79.9 [70, 93.57] | 0.443 |
| ALT, U/L | | 17 [12, 27] | 17 [12, 27] | 0.674 |
| AST, U/L | | 19 [15.25, 29.75] | 20 [15, 32.48] | 0.413 |
| PLT, 10^9/L | | 159.5 [108.25,216.75] | 159 [111.54, 204.44] | 0.56 |
| WBC, 10^9/L | | 7.42 [5.85, 9.77] | 7.66 [5.93, 10.44] | 0.25 |
| eGFR, ml/min | | 86.42 [72.25, 96.62] | 85.49 [70.09, 96.45] | 0.372 |
| LDH, U/L | | 194.5 [169.25,229.75] | 199 [172,248] | 0.182 |
| CK-MB, ng/ml | | 1.8 [1.3, 2.9] | 2 [1.4, 3.1] | 0.205 |
| TnI, ng/ml | | 1.85 [1.07, 2.73] | 2.2 [1.2, 2.9] | 0.143 |
| BNP, pg/ml | | 243 [113.75, 492.87] | 264 [103,492.87] | 0.975 |
| **Preoperative echocardiographic data** | | | | |
| LVEF, % | | 58 [51, 62] | 56.91 [51, 62] | 0.779 |
| E/A ratio | | 1.23 [0.73, 1.45] | 1.2 [0.75, 1.41] | 0.901 |
| LVDD, mm | | 51 [47, 56] | 50 [45, 56] | 0.138 |
| LVDS, mm | | 35 [30, 39] | 34 [30, 40] | 0.681 |
| **CABG data** | | | | |
| **LIMA usage (%)** | | 48.0 (27.8) | 45.8 (26.6) | 0.773 |
| **Graft number** | | 3.00 [2.00, 4.00] | 2.00 [1.00, 3.00] | <0.001 |
| **Types of valve surgery** | | | | |
| **Single valve surgery** | |  |  |  |
|  | **Mitral valve replacement (%)** | 36.0 (20.8) | 25.3 (14.6) | 0.048 |
|  | **Mitral valve repair (%)** | 24.0 (13.9) | 27.3 (15.8) | 0.529 |
|  | **Aortic valve replacement (%)** | 44.0 (25.4) | 53.7 (31.0) | 0.151 |
|  | **Aortic valve repair (%)** | 1.0 (0.6) | 0.7 (0.4) | 0.779 |
|  | **Tricuspid valve replacement (%)** | 0 (0.0) | 0 (0.0) | 1 |
|  | **Tricuspid valve repair (%)** | 5.0 (2.9) | 4.7 (2.7) | 0.897 |
| **Double valve surgery** | |  |  |  |
|  | **Double valve replacement (%)** | 3.0 (1.7) | 3.1 (1.8) | 0.965 |
|  | **Double valve repair (%)** | 1.0 (0.6) | 0.5 (0.3) | 0.547 |
|  | **Combined**  **repair and replacement (%)** | 29.0 (16.8) | 21.5 (12.5) | 0.136 |
| **Triple valve surgery** | |  |  |  |
|  | **Triple valve replacement (%)** | 0.0 (0.0) | 0.1 (0.1) | 0.318 |
|  | **Triple valve repair (%)** | 0.0 (0.0) | 0.5 (0.3) | 0.158 |
|  | **Combined**  **repair and replacement (%)** | 30.0 (17.3) | 35.4 (20.5) | 0.353 |
| **Mechanical valve (%)** | | 55.0 (31.8) | 68.3 (39.5) | 0.062 |
| **Bioprosthetic valve (%)** | | 87.0 (50.3) | 71.9 (41.6) | 0.039 |

Data are presented as median [25th-75th percentiles] or n (%). BMI, body mass index; COPD, chronic obstructive pulmonary disease; CKD, chronic kidney disease; PCI, percutaneous coronary intervention; TG, triglyceride; TC, total cholesterol; Cr, creatinine; ALT, alanine aminotransferase; AST aspartate transaminase; LDH, lactate dehydrogenase; PLT, platelet; WBC, white blood cell; eGFR, estimated glomerular filtration rate; LDH, lactate dehydrogenase; CK-MB, creatine kinase MB; TnI, troponin I; BNP, brain natriuretic peptide; LVEF, left ventricular ejection fractions; E/A, ratio early to late diastolic transmitral flow velocity; LVDD, left ventricular end-diastolic dimension; LVDS, left ventricular end-systolic dimension; CABG, coronary artery bypass grafting; LIMA, left internal mammary artery.

Table S3. Postoperative outcomes between OPCABG group and ONCABG group after PSM

|  | Unmatched | | | 1:1 PSM | | |
| --- | --- | --- | --- | --- | --- | --- |
|  | OPCABG  (n=173) | ONCABG  (n=711) | p value | OPCABG  (n=173) | ONCABG  (n=173) | p value |
| In-hospital mortality (%) | 9 (5.2) | 28 (3.9) | 0.594 | 9 (5.2) | 8 (4.6) | 1.00 |
| POAF (%) | 51 (29.5) | 281 (39.5) | 0.018 | 51 (29.5) | 70 (40.5) | 0.042 |
| Stroke (%) | 4 (2.3) | 21 (3.0) | 0.841 | 4 (2.3) | 7 (4.0) | 0.54 |
| AKI (%) | 25 (14.5) | 150 (21.1) | 0.063 | 25 (14.5) | 39 (22.5) | 0.072 |
| PMI (%) | 2 (1.2) | 9 (1.3) | 1.00 | 2 (1.2) | 0 (0.0) | 0.478 |
| CK-MB, ng/ml  24 h  48 h  72 h | 69 [43,98]  47 [28,78]  27.5 [10.1,54.1] | 66 [42.8,102.75]  45.5 [25.60,78]  22.9 [10.35,48.7] | 0.879  0.544  0.483 | 69 [43, 98]  47 [28,78]  27.5 [10.1, 54.1] | 70 [42, 108]  44 [25.8,78.7]  20.5 [8.6, 43.1] | 0.962  0.71  0.093 |
| hsTnI, ng/ml  24 h  48 h  72 h | 6.2 [3.73,9.59]  3.73 [2.05,6.44]  2.11 [0.98,3.51] | 6.2 [3.42,10.2]  3.85 [1.96,6.43]  2.89 [1.13,3.51] | 0.835  0.667  0.293 | 6.2 [3.73,9.59]  3.73 [2.05,6.44]  2.11 [0.98,3.51] | 6.19 [3.58, 10]  3.78 [2.05, 7.09]  3.51 [1.27, 3.51] | 0.767  0.436  0.054 |
| Operation time ,hours | 6 [5, 7] | 6 [5, 7] | 0.587 | 6 [5, 7] | 6 [5, 7] | 0.221 |
| Crossclamp time ,min | 86 [69, 122] | 113.50 [93.25, 140] | 0.001 | 86 [69, 122] | 111 [92, 133] | 0.001 |
| CPB time, min | 160 [120, 189] | 168 [143.25, 204] | 0.001 | 160 [120, 189] | 166 [142, 204] | 0.002 |
| Ventilation time, hours | 25 [18, 55.5] | 23.50 [17.50, 49] | 0.393 | 25 [18, 55.5] | 23 [17.50, 58] | 0.54 |
| LOS, days | 8 [6, 10] | 8 [6, 10] | 0.728 | 8 [6, 10] | 8 [6, 10] | 0.692 |
| ICU time of stay , hours | 42 [19, 68] | 35 [18, 68] | 0.402 | 42 [19, 68] | 37 [18, 70] | 0.531 |
| IABP (%) | 13 (7.5) | 66 (9.3) | 0.56 | 13 (7.5) | 18 (10.4) | 0.451 |
| ECMO (%) | 2 (1.2) | 13 (1.8) | 0.775 | 2 (1.2) | 4 (2.3) | 0.68 |

Data are presented as median [25th–75th percentiles] or n (%). POAF, postoperative atrial fibrillation; AKI, acute kidney injury; PMI, postoperative myocardial infarction; CK-MB, creatine kinase MB; hsTnI, high-sensitivity troponin I; CPB, cardiopulmonary bypass; LOS, length of stay; ICU, intensive care unit; IABP, intra-aortic ballon pump; ECMO, extracorporeal membrane oxygenator.
